# Ti Dopants as a Morphology-Stabilizing Agent in Mesoporous Ruthenium Oxide Electrodes

**DOI:** 10.1021/acs.inorgchem.5c01962

**Published:** 2025-10-20

**Authors:** Nipon Deka, Denis Bernsmeier, Rik Mom

**Affiliations:** † Leiden Institute of Chemistry, 201229 Leiden University, PO Box 9502, 2300 RA Leiden, The Netherlands; ‡ Department of Inorganic Chemistry, Fritz-Haber Institute of the Max-Planck Society, 14195 Berlin, Germany; § Institut für Chemie, 26524 Technische Universität Berlin, Str. des 17. Juni 124, 10623 Berlin, Germany

## Abstract

Due to its excellent electrochemical properties, ruthenium oxide is used in applications ranging from electrocatalysis to sensors and energy storage. However, the scarcity and cost of ruthenium demand strategies for maximizing its efficient use. Primary tools in this are nanostructuring, which enhances the accessible surface area, and crystallization, which improves stability against dissolution. However, these two strategies are typically in conflict: crystallization via high-temperature calcination often leads to pore collapse in nanostructured RuO_*x*_. To deal with this trade-off between porosity and stability, here, we demonstrate that titanium doping stabilizes the mesoporous structure of RuO_*x*_ during high-temperature calcination, enabling the synthesis of highly crystalline yet mesoporous RuTiO_*x*_ films. Unlike earlier Ti-doped RuO_*x*_ systems, which often suffer from a loss of porosity or structural cracking at elevated temperatures, our soft-templated RuTiOx films retain a well-defined mesostructure and exhibit strong electronic interaction between Ti–O and Ru–O bonds. Electrochemical testing shows that the RuTiO_*x*_ electrodes exhibit high catalytic activity for the chlorine evolution reaction (CER), achieving current densities above 500 mA cm^–2^ at 1.50 V_Ag/AgCl_ in 3 M HCl, with improved operational stability compared to pure RuO_*x*_. This work highlights a dual role of Ti: stabilizing film morphology and tuning the electronic structure, offering a general strategy to combine high surface area with long-term durability in mesostructured electrocatalysts.

## Graphical abstract

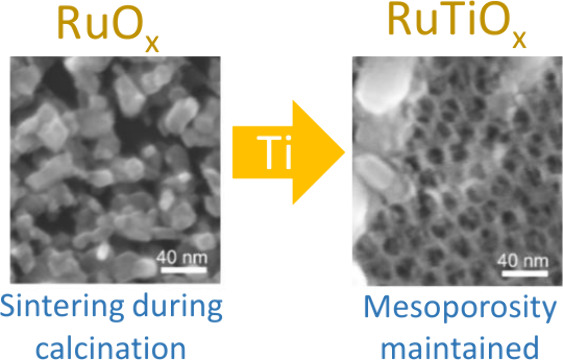

## Introduction

Ruthenium oxide is a cornerstone material in electrochemistry, with diverse applications such as chlorine and hydrogen production,^1^ wastewater treatment,^2,3^ batteries beyond Li-ion,^4,5^ supercapacitors,^6^ and electrochemical sensors.^7^ Most prominent among these applications is the use of RuO_2_-based dimensionally stable anodes (DSAs) for the chlorine evolution reaction (CER), a key component of the industrial chlor-alkali process and wastewater treatment.^8^ The high performance of RuO_2_-DSAs in the CER is attributed to their high electrical conductivity, excellent corrosion resistance, and remarkable catalytic activity in acidic and neutral chloride-containing electrolytes. The synergy between RuO_2_ and other oxides (such as TiO_2_, SnO_2_, and IrO_2_) improves electrode stability and reduces Ru dissolution, thereby extending electrode lifespan.^9–11^ Recent advancements in material engineering, including dopant incorporation and surface modifications, continue to refine RuO_2_-based DSAs, balancing activity and durability.^12^ These developments reinforce their role as leading candidates for sustainable and cost-effective chlorine production technologies.

Despite these advances, the high cost and limited availability of ruthenium push a continued striving to further maximize its efficiency. A promising approach to achieve this is through nanostructuring techniques^13–19^ that create porous electrodes with a high catalytically active surface area. For example, synthesizing mesoporous oxide films using micelle-templated methods offers a bottom-up approach to induce three-dimensional mesoporosity, a strategy that has proven effective for various transition metal oxides^20–29^ like TiO_2_, Nb_2_O_5_, WO_3_, Fe_2_O_3_, NiO_*x*_, IrO_*x*_, and IrTiO_*x*_. This soft-templated nanostructuring approach has also been used to create high surface-area ruthenium oxides.^30^ However, critical challenges are the sintering, pore collapse, and cracking of the mesoporous oxide films during calcination^13,30–33^ Including such a calcination step in the synthesis is critical for the preparation of high-performance ruthenium oxide electrocatalysts because it crystallizes the oxide lattice, which reduces the dissolution of ruthenium under oxidizing electrocatalytic conditions.^34^ However, the atomic mobility of Ru and O atoms that facilitates this crystallization also triggers sintering and densification, resulting in a significant loss in surface area and pore volume. For example, Song et al.^35^ reported a grain size increase from ∼2 nm at 200 °C to 8 nm at 400 °C and 60 nm at 600 °C with a corresponding surface area loss of a factor ∼20. Similar results were found by Malmgren et al.,^36^ who found a 4.6× surface area decrease when increasing the calcination temperature from 350 to 550 °C to improve the crystallinity of their catalysts. These examples underscore the inherent challenge of maintaining mesoporosity during thermal treatments aimed at enhancing stability.

To address the challenge of creating a crystalline ruthenium-based oxide while preventing the collapse of the nanostructure during high-temperature calcination, we explored the possibility of introducing a third element into the RuO_*x*_ lattice through doping. So far, this doping strategy has been used to stabilize the oxide lattice under electrocatalytic conditions.^11,37–39^ Here, we investigated whether doping could also be a viable approach to stabilize the porous structure of ruthenium oxide during high-temperature calcination. We specifically focus on Ti as the dopant, because it is abundant, stable under oxidizing conditions,^43,44^ and it offers a good lattice match with rutile RuO_2,_ ^45,46^ making it an ideal stabilizing agent, as supported by prior studies.^47,48^ By contrast, dopants such as Mn, Sn, or Ir often introduce challenges like high cost, phase segregation, and lattice strain, the latter two of which may compromise long-term stability.^40–42^

We show that Ti doping can indeed serve as a calcination-stabilizing agent without compromise on activity and stability. Key to our synthetic strategy is that the Ti and Ru are coprecipitated into a soft-templated mesoporous precursor structure prior to calcination. Our results show that this prevents the crack formation and loss of well-defined porosity at high annealing temperatures observed in earlier work on Ru–Ti oxides.^13,31–33^ Structural and spectroscopic analyses show that the resulting materials have a crystalline, well-defined mesoporous structure with a mixed Ru–Ti composition. X-ray photoelectron spectroscopy (XPS) and X-ray absorption spectroscopy (XAS) revealed that Ti modifies the electronic structure at the Ru and oxygen sites, specifically influencing the covalency of the Ru–O bond. Importantly, chlorine evolution activity tests showed that Ti doping favors both high activity and stability.

## Methods

### Oxide Film Synthesis

The mesoporous oxide films were prepared via evaporation-induced self-assembly (EISA) using a simple dip-coating procedure, analogous to previous work on IrTiO_x._ ^28^ We selected the triblock copolymer as the structure-directing agent due to its superior micelle-forming characteristics and structural stability compared to more commonly used pluronic-type copolymers.^49–52^ Its strong hydrophilic–hydrophobic contrast and enhanced micelle stability facilitate better mesostructure control and improve thermal robustness—a crucial factor for preserving porosity during the high-temperature crystallization of metal oxides required in our synthesis.

A PEO_213_-PB_184_-PEO_213_ block copolymer (PEO = poly­(ethylene oxide), PB = polybutadiene, containing 18,700 g mol^–1^ PEO and 10,000 g mol^–1^ PB, source: Polymer Service Merseburg GmbH) was used as the template polymer. As reported previously,^53^ thermogravimetric analysis (TGA) shows that the employed block copolymer PEO_213_–PB_184_–PEO_213_ starts to decompose at temperatures around ∼250 °C and rapidly decomposes between 350 and 425 °C, indicating a better thermal stability than common pluronic-type polymers. The observed thermal stability of PEO-PB-PEO is similar to the stability of poly­(ethylene-*co*-butylene)-*b*-poly­(ethylene oxide) (KLE), which is well-known for its templating capabilities.

Two separate dispersions were prepared for RuO_*x*_ and RuTiO_*x*_. For the RuO_*x*_ films, 67.3 mg of PEO_213_-PB_184_-PEO_213_ block copolymer was dissolved in 3.95 mL of EtOH at 45 °C while stirring for 1 h. To this dispersion, 120.0 mg of RuCl_3_.n H_2_O (Alfa Aesar, 99.9%, 38% Ru min) was subsequently added, followed by another 30 min stirring at 45 °C. Similarly, for the RuTiO_*x*_ films, 56 mg of PEO_213_-PB_184_-PEO_213_ block copolymer was dissolved in 2.3 mL of EtOH at 45 °C while stirring for 1 h. Next, a solution containing 4.4 mL of EtOH and 100 μL of titanium­(IV) chloride (TiCl_4_, Sigma-Aldrich, 99.9%) was prepared. **Caution!** TiCl_4_ is highly toxic and reactive. It should be stored under an inert gas and handled with care. TiCl_4_ reacts with atmospheric humidity to produce a vapor of HCl and Ti-containing compounds. TiCl_4_ also reacts exothermically with ethanol. During handling, air exposure should be minimized by, e.g., using a syringe to take out the liquid TiCl_4_ from the container. It should be added dropwise into the solvent while stirring. Protective equipment including safety glasses must be worn.

1.1 mL portion of the solution was added dropwise to the polymer solution under stirring. Finally, 57.7 mg of RuCl_3_·*n*H_2_O (Alfa Aesar, 01104, 38 wt % Ru) was added to the solution followed by another 30 min stirring at 45 °C. The final coating solution contained 0.22 mmol of Ru and 0.23 mmol of Ti. From EDX analysis, we have found a linear correlation between the Ru:Ti molar ratio in the coating solution and that in the final catalyst film.

To ensure reproducible dip-coating conditions, the dispersion was transferred into a Teflon cuvette heated to 45 °C in a controlled air environment (20 °C and 45% relative humidity). The desired substrate was mounted on a z-drive, dipped into the dispersion, and then withdrawn at 300 mm/min. This dipping procedure leaves a dispersion film on the electrode, which self-assembles into a mesoporous structure via micelle formation while drying (EISA). The fully withdrawn substrate was dried in air for at least 5 min. Finally, the coated substrates were calcined for 10 min in air at various temperatures in a preheated muffle furnace to remove the PEO_213_–PB_184_–PEO_213_ sacrificial soft template and reach different levels of oxide crystallinity. The reproducibility of the procedure was confirmed using repeated synthesis and analysis of the catalyst films.

## Characterization

### Scanning Electron Microscopy (SEM)

SEM images were obtained by using a JEOL 7401F instrument with an accelerating voltage of 10 kV.

### Transmission Electron Microscopy (TEM)

TEM and selected area electron diffraction (SAED) images were obtained with an FEI Tecnai G2 20 S-Twin at an accelerating voltage of 200 kV. The film samples were scraped off from Si wafers and then collected on a TEM-grid.

### X-ray Diffraction (XRD)

Respective oxide films were coated on polished silicon wafers, and XRD patterns were recorded with a Bruker D8 Advance instrument using Cu Kα radiation.

### X-ray Spectroscopy

The XPS and total electron yield X-ray absorption spectroscopy (TEY-XAS) measurements were conducted in high vacuum at the ISISS beamline at the BESSY II electron storage ring operated by Helmholtz-Zentrum Berlin für Materialien and Energie. Subsequent scans were monitored to ensure the absence of beam damage effects.

### Electrochemical Measurements

The chlorine evolution activity and stability of the mesoporous films were measured via cyclic voltammetry and chronopotentiometry, respectively, in a flow cell with a three-electrode setup which employs a Ag/AgCl reference electrode and a platinum wire as the counter electrode. The electrolyte used was aqueous 3 M HCl. To prepare the working electrode, a Ti substrate was polished by SiO_2_-polishing paste (Buehler, MasterMet 2, noncrystallizing colloidal silica suspension, 0.02 mm), then ultrasonicated in water, and rinsed with ethanol (VWR Chemicals, 99.98% absolute), followed by dip coating as described in the film preparation section.

### Electrical Conductivity

Electrical conductivity was measured for catalytic coatings on microscope slides with a Keithley Model 6517B electrometer employing an 8 × 8 pin probe head with an altering polarity sequence of the pins (2-point probe). Light-induced enhancement of electrical conductivity observed for semiconducting materials (e.g., TiO_2_) was avoided by measuring the material in a dark environment. The applied potential was varied between 50 and 450 mV and was increased by a factor of 1.2 for each step. Each point was measured for about 8 s. The conductivity was calculated via Ohm’s law using a device-specific correction factor derived from the measurement of a commercial indium tin oxide layer on glass. At least three different positions on each coating were measured.

## Results

To assess the effects of titanium doping in mesoporous ruthenium oxides, we synthesized a series of mesoporous RuO_*x*_ and RuTiO_*x*_ oxides employing various calcination temperatures (details in the Methods section). The morphology and crystallinity of the films were investigated by using scanning electron microscopy (SEM), transmission electron microscopy (TEM), and X-ray diffraction (XRD). As shown in Figure 1, calcination temperatures below 350 °C universally leads to a very open mesoporous structure with a typical pore diameter of 15–20 nm. Similar to previous studies^27,28,54^ using the PEO-PB-PEO amphiphilic triblock copolymer template, the pores are stacked in a disordered array. Cross-section SEM measurements (Figure S1a,b in the Supporting Information) indicated that a film thickness of 60–90 nm is reached with a single dip. Thicker films up to 300 nm can readily be created by cycles of dipping and 10 min of calcination at the respective temperature, without apparent change in the film properties (Figure S1c in the Supporting Information). Large-scale SEM images (Figure S2 in the Supporting Information), TEM micrographs (Figures S3 and S4 in the Supporting Information), and optical inspection showed that the films produced here are highly homogeneous and free of cracks and open spaces, which protects the Ti substrate underneath.

**Figure 1:**
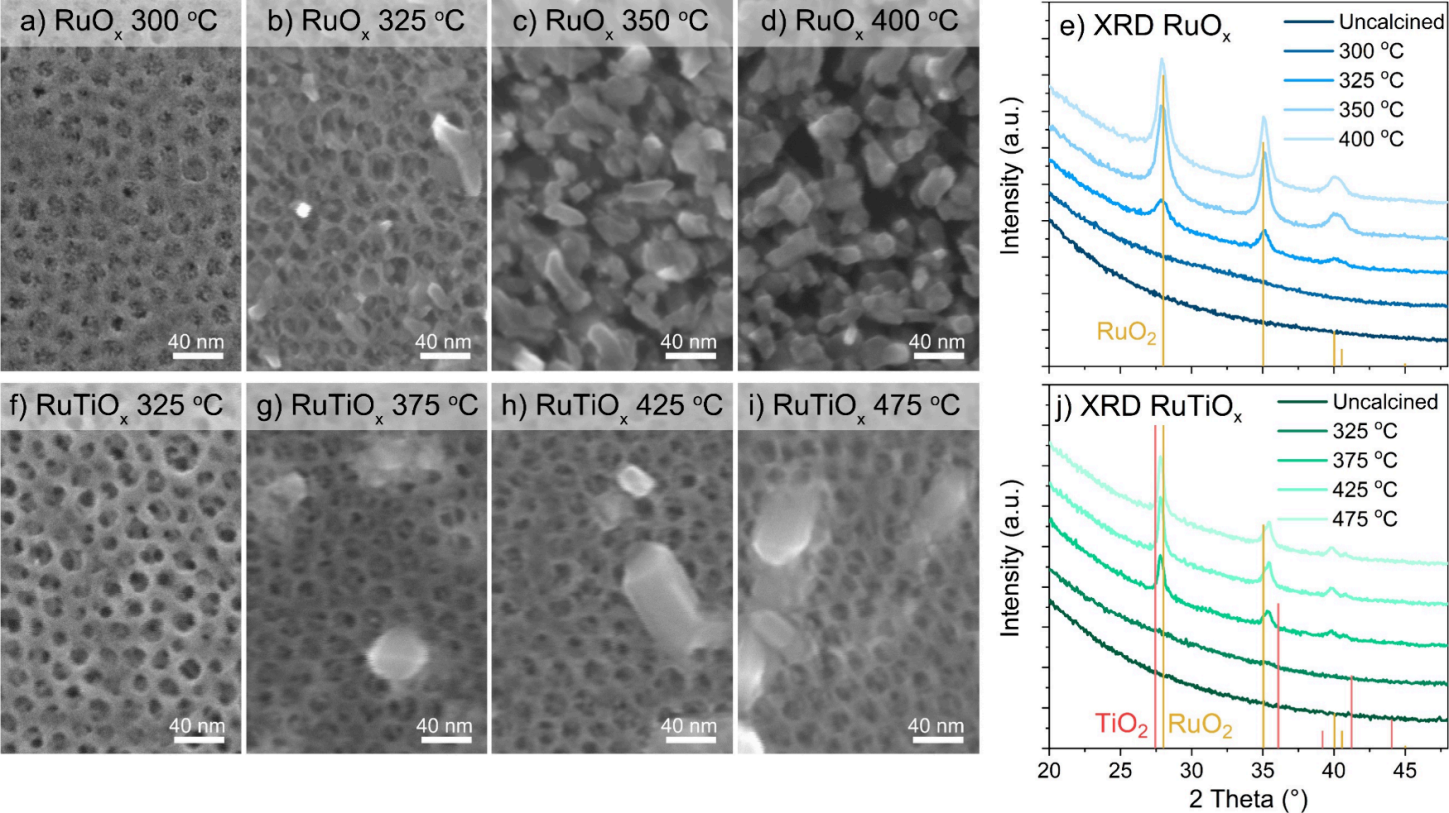
(a–i) Morphology of the as-prepared mesoporous RuO_*x*_ and RuTiO_*x*_ films as observed under SEM. The XRD patterns in (e) and (j) are compared to rutile RuO_2_ (PDF 00-040-129) and TiO_2_ (PDF 21-1276).

The employed calcination temperature has a marked effect on the film morphology and crystallinity. For RuO_*x*_, the mesoporous structure collapses once the film makes the transition from an amorphous structure to the rutile crystal phase at temperatures >350 °C. This collapse does not occur for RuTiO_*x*_, which retains the same pore structure even when full crystallization of the film is reached. Hence, it appears that the addition of Ti has a stabilizing effect on the film morphology. From the XRD patterns, it is also clear that the crystal structure of the material is affected by the presence of Ti. As shown in Figure 1j, the lattice spacing in the RuTiO_*x*_ films lies between that of RuO_2_ and TiO_2_, suggesting the formation of a homogeneously mixed oxide with properties that differ from its pure constituents. However, the formation of some larger crystallites on the surface of the films at high temperatures does indicate that some demixing occurs, in agreement with literature observations^33,55^ and supported by analysis of the (near-)­surface Ru:Ti composition using XPS (SI Section 4). The demixing may be attributed to stress in the RuTiO_*x*_ oxide lattice due to the difference in the Ti–O and Ru–O bond length.

The observed structure of the oxide films has a strong relation with its composition and electronic properties. This is quite apparent in the Ru 3d and O 1s peak shapes observed using XPS (see Figure 2). The amorphous or semicrystalline RuO_*x*_ films obtained from calcination below 350 °C show a broad Ru 3d doublet at a binding energy slightly higher than for rutile RuO_2_, which is typical for hydrous ruthenium­(IV) oxide.^56,57^ Once the oxide crystallizes at a higher calcination temperature, the satellite peaks typical for rutile RuO_2_ become apparent in the spectrum. Concomitantly, with increasing calcination temperature, a decreasing OH shoulder is observed in the O 1s spectra at 530.5 eV. Note, however, that there is also a contribution from carbon species in both the Ru 3d and O 1s spectra. For the O 1s spectra, such C–O species appear around 531–532 eV, while in the Ru 3d spectrum, the Ru 3d_3/2_ peak is broadened and pushed up by underlying C 1s contributions in the range of 284–288 eV.

**Figure 2:**
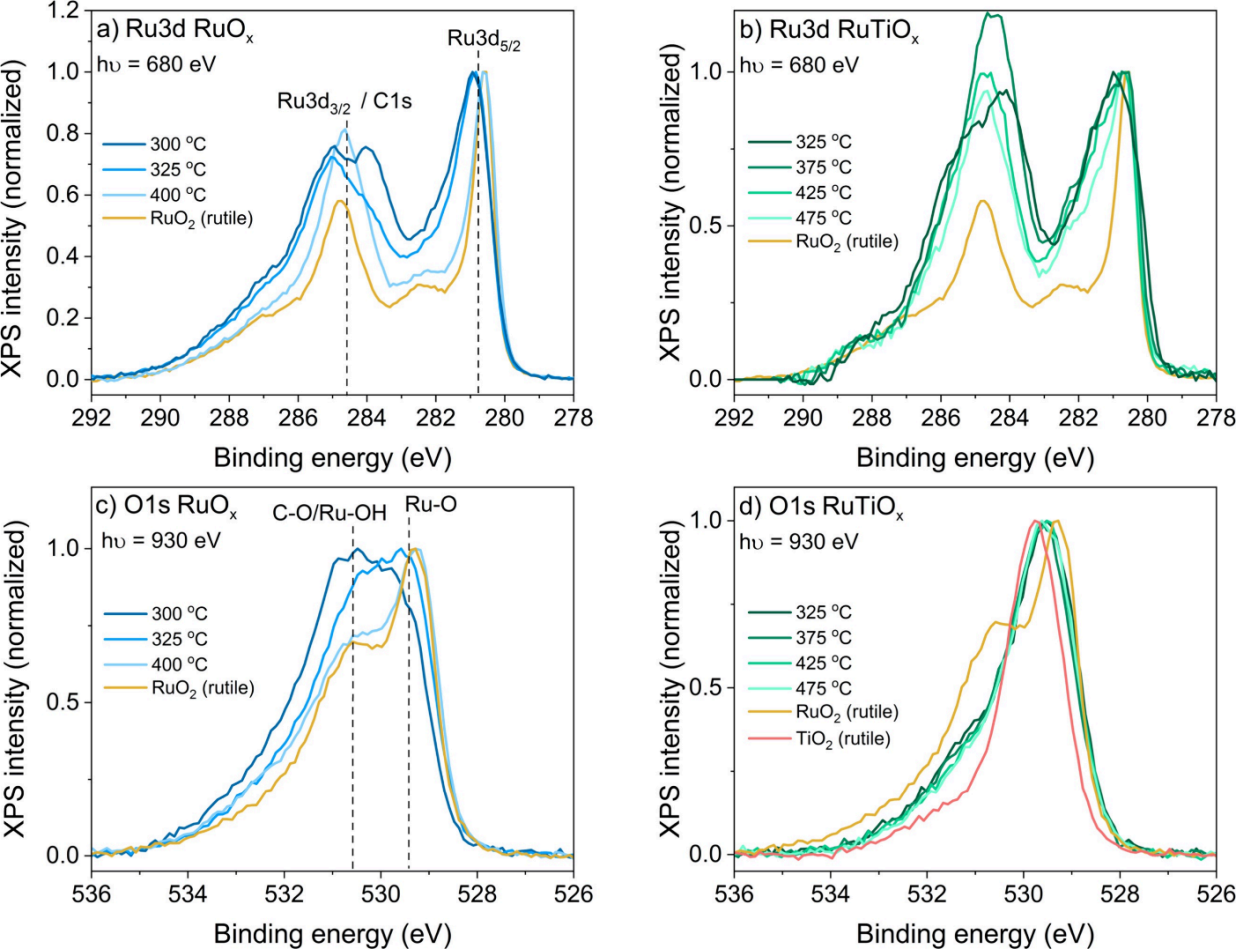
Electronic structure of the as-prepared mesoporous RuO_*x*_ and RuTiO_*x*_ films. (a) Ru 3d/C 1s spectra of RuO_*x*_. (b) Ru 3d/C 1s spectra of RuTiO_*x*_. (c) O 1s spectra of RuO_*x*_. (d) O 1s spectrum of RuTiO_*x*_.

The RuTiO_*x*_ films show marked differences in electronic structure with respect to the RuO_*x*_ films. For pure RuO_*x*_, the film has semimetallic properties at low calcination temperature, as evidenced by their low conductivity (Figure 3) and the broad, high binding energy Ru 3d doublet (Figure 2b) that results from poor final state screening by the valence electrons (described in Section 6 of the Supporting Information). At high calcination temperatures, the (crystalline) RuO_*x*_ films attain metallic properties typical for rutile RuO_2_. For the RuTiO_*x*_ film, this is not the case: even the crystalline RuTiO_*x*_ films produced at high temperature show semimetallic conductivity and a broad Ru 3d doublet. This impact of Ti on the electronic structure is also reflected in the O1s lattice O peak, which lies between the typical values for TiO_2_ and RuO_2_. This shows that the Ru–O and Ti–O bonds affect each other. The nature of this effect can be further resolved using the O K-edge absorption spectra (Figure 4), which show separate resonances for the Ru–O and Ti–O bonds. Compared to those of pure RuO_2_ and TiO_2_, these resonances are broadened, again indicating interactions between the Ru–O and Ti–O hybridized states. The most pronounced effect of these interactions is the repression of Ru–O resonance, indicative of a more complete filling of the Ru 3d-O 2p hybridized states (detailed analysis in Section 7 of the Supporting Information). This can be interpreted as an increase in the ionicity of the Ru–O bond; i.e., the Ru–O bond appears to “borrow” some of the Ti–O bond’s strong ionicity in the mixed Ru–Ti oxide lattice. Overall, the analysis from Figures 2–4 thus indicates that installing Ti in the mesoporous ruthenium oxide creates a mixed RuTiO_*x*_ phase with a more ionic character than pure RuO_2_, in line with theoretical studies on Ru–Ti oxides.^61–63^

**Figure 3:**
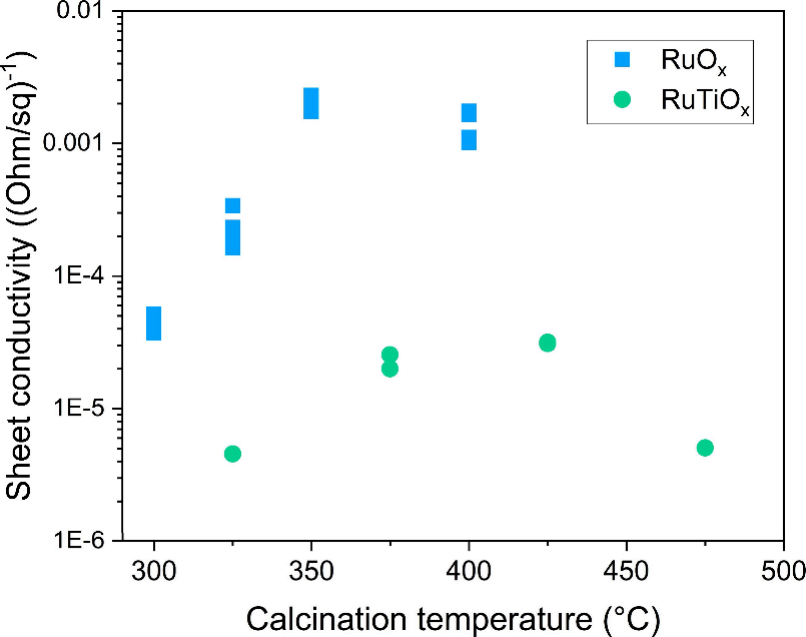
Conductivity of the as-prepared oxide films.

**Figure 4:**
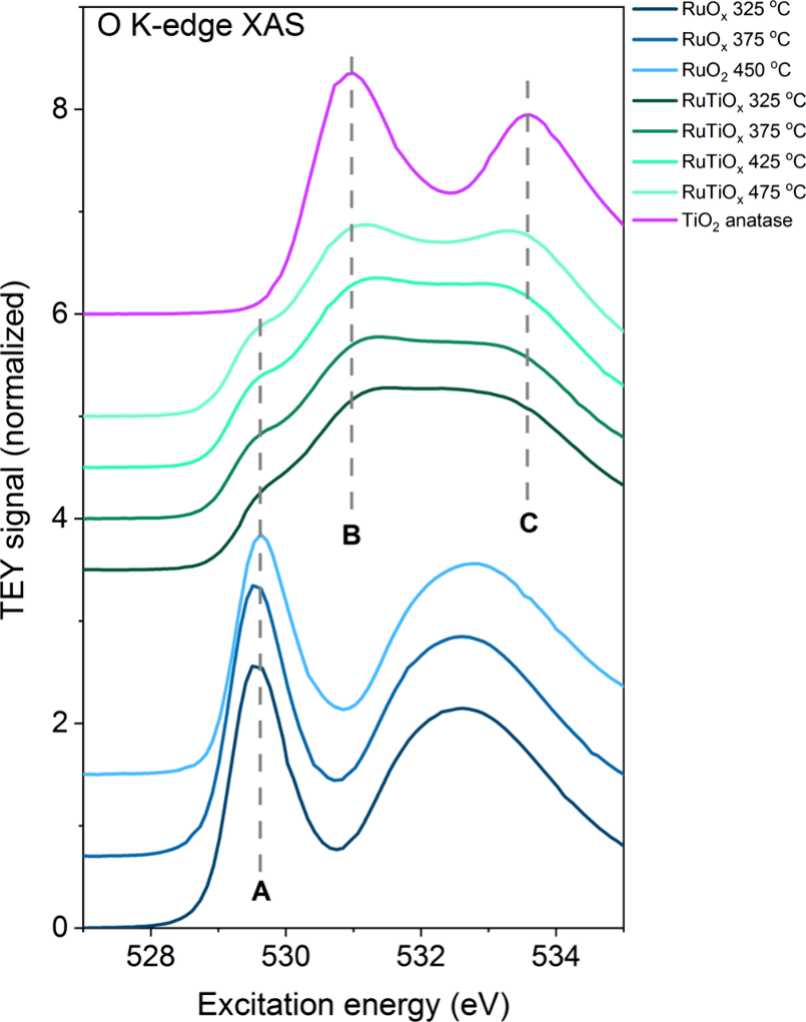
O K-edge XAS of RuO_*x*_ and RuTiO_*x*_ films. The spectra are normalized to the edge jump at 550 eV. A,B, and C designate the main resonances of the Ru–O bond (A) and Ti–O bond (B,C).

To also evaluate the electronic structure from the perspective of the Ti atoms in the RuTiO_*x*_ lattice, we recorded the Ti L-edge XAS spectra (Figure 5). As a reference, we synthesized TiO_*x*_ using the same soft-template synthesis (Figure 5a). The Ti L-edge XAS consists of contributions from L_3_ (Ti 2p_3/2_ → 3d) and L_2_ (Ti 2p_1/2_ → 3d) transitions. These bands split into t_2g_ and e_g_ features because of the TiO_6_^8–^ octahedral ligand field.^65^ For fully crystallized mesoporous TiO_2_ (calcined at 475 °C), we observe that the e_g_ feature at the L_3_ edge further splits, which is the result of distortion in the octahedral ligand coordination^70^·^71^ The strong asymmetric split observed here is a fingerprint of the anatase crystallographic phase,^66,67^ showing that in our synthesis, pure TiO_*x*_ tends to crystallize into an anatase structure with the increasing in calcination temperature.

**Figure 5:**
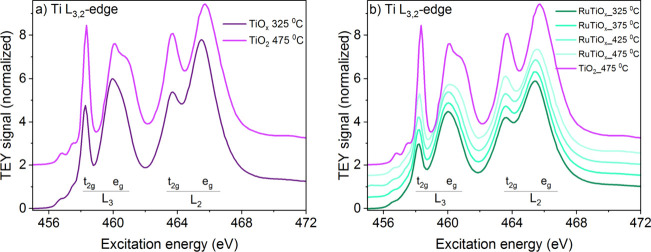
Ti L_3,2_-edge spectra of mesoporous (a) TiO_*x*_ and (b) RuTiO_*x*_ films synthesized via EISA.

Comparing the Ti L-edge of the RuTiO_*x*_ samples (Figure 5b) to that of the anatase TiO_2_ reference, we see two notable differences. First, at the L_3_-edge, the RuTiO_*x*_ samples display a t_2g_ peak lower in intensity relative to the e_g_ peak. This indicates that titanium in the RuTiO_*x*_ lattice has a less ionic character than in pure TiO_*x*_, consistent with the transfer of ionic character from Ti to Ru inferred from the O 1s and Ru 3d spectra in Figure 2. Second, the lack of splitting in the e_g_ peak of the L_3_-edge indicates a high degree of symmetry in the octahedral coordination of Ti in RuTiO_*x*_. First, this means that the anatase crystal structure is not stabilized in RuTiO_x,_ ^68,69^ consistent with the XRD results indicating a mixed RuTiO_*x*_ rutile phase. Second, it implies that the strain-induced distortion in the oxide lattice due to Ti–Ru mixing is modest, consistent with the relatively good lattice match between Ti and rutile RuO_2_ ^45,46^

Overall, the analysis of the (electronic) structure above provides two insights. (1) The RuTiO_*x*_ is a properly mixed Ru–Ti oxide with significant interactions between the Ru–O and Ti–O bonds. (2) Introducing Ti into the RuO_*x*_ lattice allows the Ru–O bonds to “borrow” some of Ti–O bond’s properties, resulting in a more ionic Ru–Ti oxide compared to pure RuO_2_. We hypothesize that this effect is key to the remarkable stability of the mesoporous RuTiO_*x*_ structure during calcination, in combination with the high-temperature stability of the chosen PEO-PB-PEO soft template. Both of these factors indirectly control the mobility of the Ru­(Ti) and O atoms: higher ionicity may induce rigidity in the oxide lattice, while the persistence of the template during the first minutes of calcination provides an additional barrier for restructuring. Indeed, the combination of a highly ionic lattice and this same template also endow pure mesoporous TiO_2_ with a very high stability against sintering, allowing it to be readily crystallized without loss of the mesoporous structure.^49,60^

For a full picture of the final mesoporous catalyst, it is also important to analyze minority species in the lattice that are introduced by the chosen synthesis method. In our case, these are chloride ions originating from the RuCl_3_ precursor and carbon-based remnants originating from the PEO-PB-PEO soft template. From Cl 2p spectra (Figure S6 in the Supporting Information), it is observed that indeed a small amount of Cl ions is built into the lattice, which is often observed for ruthenium oxides prepared from a RuCl_3_ precursor.^57–59^ Note that we do not observe any unreacted RuCl_3_, as evidenced by TEM and XRD. Analysis of the Cl content shows that it is rather similar for RuO_*x*_ and RuTiO_*x*_. Along the same lines, we find that the carbon content of the RuO_*x*_ and RuTiO_*x*_ is similar (see SI Section S8). Importantly, these observations indicate that Cl and carbon remnants are not an underlying factor in the observed differences between RuO_*x*_ and RuTiO_*x*_.

To evaluate how the mesoporosity and electronic structure differences between RuO_*x*_ and RuTiO_*x*_ affect their electrocatalytic performance, we measured the activity and stability of the mesoporous oxide films during the CER (Figure 6). The cyclic voltammograms in Figure 6a provide an estimate of the electrochemically active surface area. We observe that at low calcination temperature, both RuO_*x*_ and RuTiO_*x*_ exhibit a similar surface redox current, suggesting a similar active surface area. Surprisingly, the active surface area increases for a higher calcination temperature for both RuO_*x*_ and RuTiO_x._ This can be attributed to the incomplete combustion of the polymer template below 325 °C, which may limit the access of the electrolyte to the electrode surface inside the pores. This is in line with the strong C 1s signals observed for low calcination temperatures in Figure 2. Increasing the calcination temperature crystallizes the films and leads to further removal of the polymer template. Consistent with the observed collapse of the mesoporous structure for RuO_2_ at high calcination temperature, we observe a higher active surface area for RuTiO_*x*_ compared to that of RuO_2_.

**Figure 6:**
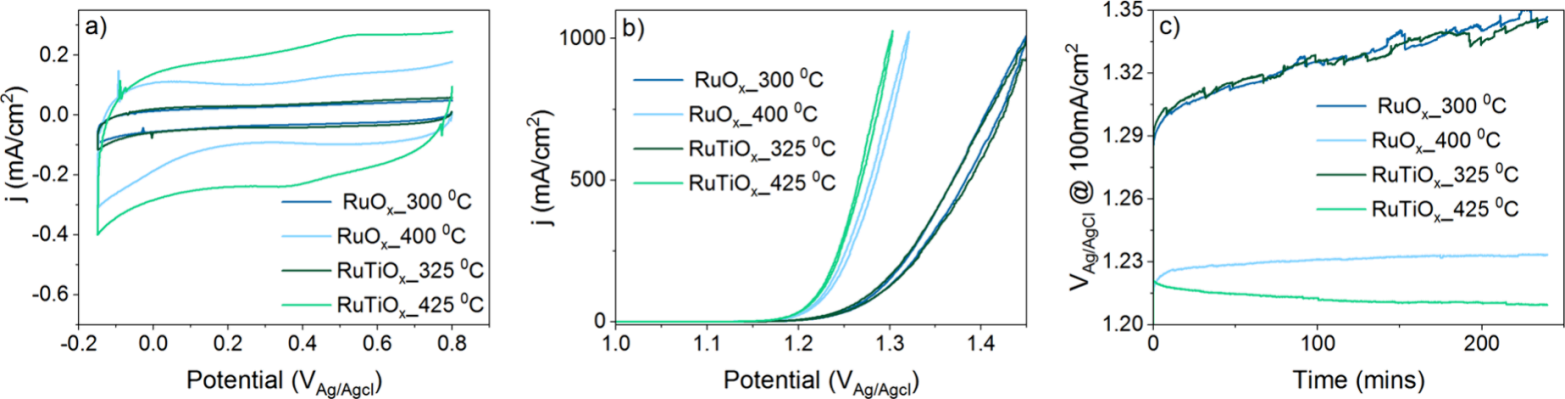
Electrochemical characterization: (a) cyclic voltammetry at 50 mV/s, (b) activity measurement at 5 mV/s, and (c) stability measurement via chronopotentiometry.

The higher surface area of RuTiO_*x*_ translates to higher chlorine evolution activity. As observed in Figure 6b, the activity appears to scale with the surface area, independent of the composition of the electrode (RuO_*x*_ vs RuTiO_*x*_). This suggests that the electronic structure differences observed for RuO_*x*_ and RuTiO_*x*_ have little effect on the catalytic performance for chlorine evolution. Importantly, it also shows that the lower conductivity of the RuTiO_*x*_ films (Figure 3) does not induce a significant *iR* drop across the film, even at high current densities. This can be attributed to the modest film thickness (∼200 nm), which ensures a short conductance path toward the more conductive Ti substrate.^72,73^

Apart from the improved activity through the RuTiO_*x*_’s higher surface area, chronopotentiometry (Figure 6c) reveals that the stability of the crystalline RuTiO_*x*_ also exceeds that of RuO_*x*_. For crystalline RuO_2_ (calcined at 400 °C), the overpotential gradually increases over the course of four h, indicating loss of active material over time. In contrast, a decreasing overpotential is observed for RuTiO_*x*_. This indicates that instead of dissolving, RuTiO_*x*_ is undergoing a transformation that further activates the material. This might be caused by the removal of carbon remnants over time which exposes more active sites. The improved stability of RuTiO_*x*_ with respect to RuO_*x*_ is in line with other studies on doped Ru oxides^9–11,37–39^ and can likely be related to its modified electronic structure (increased ionic character of the Ru–O bond). Such electronic structure modifications are believed to stabilize the Ru against overoxidation and subsequent dissolution as high-oxidation-state RuO_3_ or RuO_4_^2–^.

## Conclusion

To summarize, our work shows that introducing Ti as a dopant into mesoporous ruthenium oxides enables the formation of a high surface area crystalline ruthenium oxide electrocatalyst with high catalytic activity and stability during the CER. Using a soft-templating approach, we prepared uniform, crack-free mesoporous RuO_*x*_ and RuTiO_*x*_ films with a crystallinity that can be systematically tuned using the calcination temperature. The titanium has two effects in these films: (1) It stabilizes the mesoporous structure at high calcination temperatures and (2) it changes the electron structure of the Ru–O bond. We find that the former is the primary responsible for the high activity of the RuTiO_*x*_ films, whereas the latter appears to improve the stability of the oxide lattice.

Importantly, the RuTiO_*x*_ films deliver chlorine evolution performance that is comparable to or exceeds benchmark materials such as IrO_2_ and commercial DSAs,^72,74^ with a distinct advantage in mesostructure retention and stability under extended operation. This makes RuTiO_*x*_ a promising candidate for industrial electrolyzers seeking higher durability and reduced precious metal content. While the current synthesis employs soft-templating and dip-coating—methods that may present scale-up challenges—the compatibility with Ti substrates, short calcination times, and use of abundant precursors suggests feasible translation to scalable manufacturing processes such as spray deposition or roll-to-roll coating. Overall, our work highlights the possibility to use dopants to control not only the electronic structure but also the morphology of mesoporous oxides.

## Supplementary Material



## Notes

Additional SEM; TEM; XPS; XAS data; carbon content analysis; and a literature comparison of CER activities (PDF)
