# Supplementary material for: Ti Dopants as a Morphology-Stabilizing Agent in Mesoporous Ruthenium Oxide Electrodes
Source: Inorg Chem. 2025 Oct 20;64(43):21333–40. doi: 10.1021/acs.inorgchem.5c01962 (PMC12587398; doi:10.1021/acs.inorgchem.5c01962)
Supplement: Supplementary file 1 [file ic5c01962_si_001.pdf]

Supporting information for:

Ti dopants as a morphology-stabilizing agent in mesoporous ruthenium oxide electrodes

Nipon Deka,[1,2], Denis Bernsmeier [3], Rik Mom [1*]*

1. Leiden Institute of Chemistry, Leiden University, PO Box 9502, 2300 RA Leiden, The Netherlands
2. Department of Inorganic Chemistry, Fritz-Haber Institute of the Max-Planck Society, 14195 Berlin, Germany
3. Technische Universität Berlin, Institut für Chemie, Str. des 17. Juni 124, 10623 Berlin, Germany

*n.deka@lic.leidenuniv.nl
*r.v.mom@lic.leidenuniv.nl

1. Cross section of the oxide films

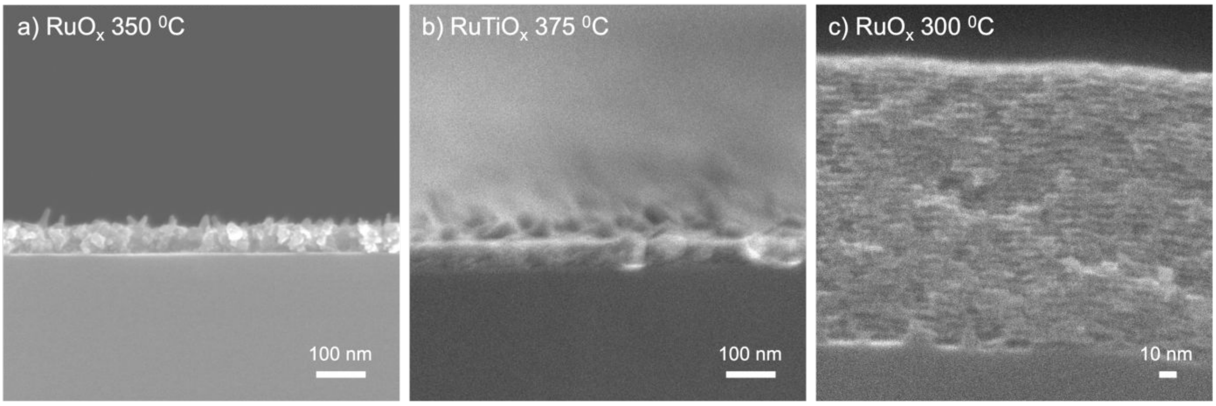

Figure S1: Cross-section SEM micrographs of the as-prepared films. a-b) Single layer dip-coating of RuO_x and $RuTiO_x$ films, c) A RuO_x film synthesized by 2-layer dip coating resulting in a film thickness of approximately 160 nm.

2. Large scale morphology

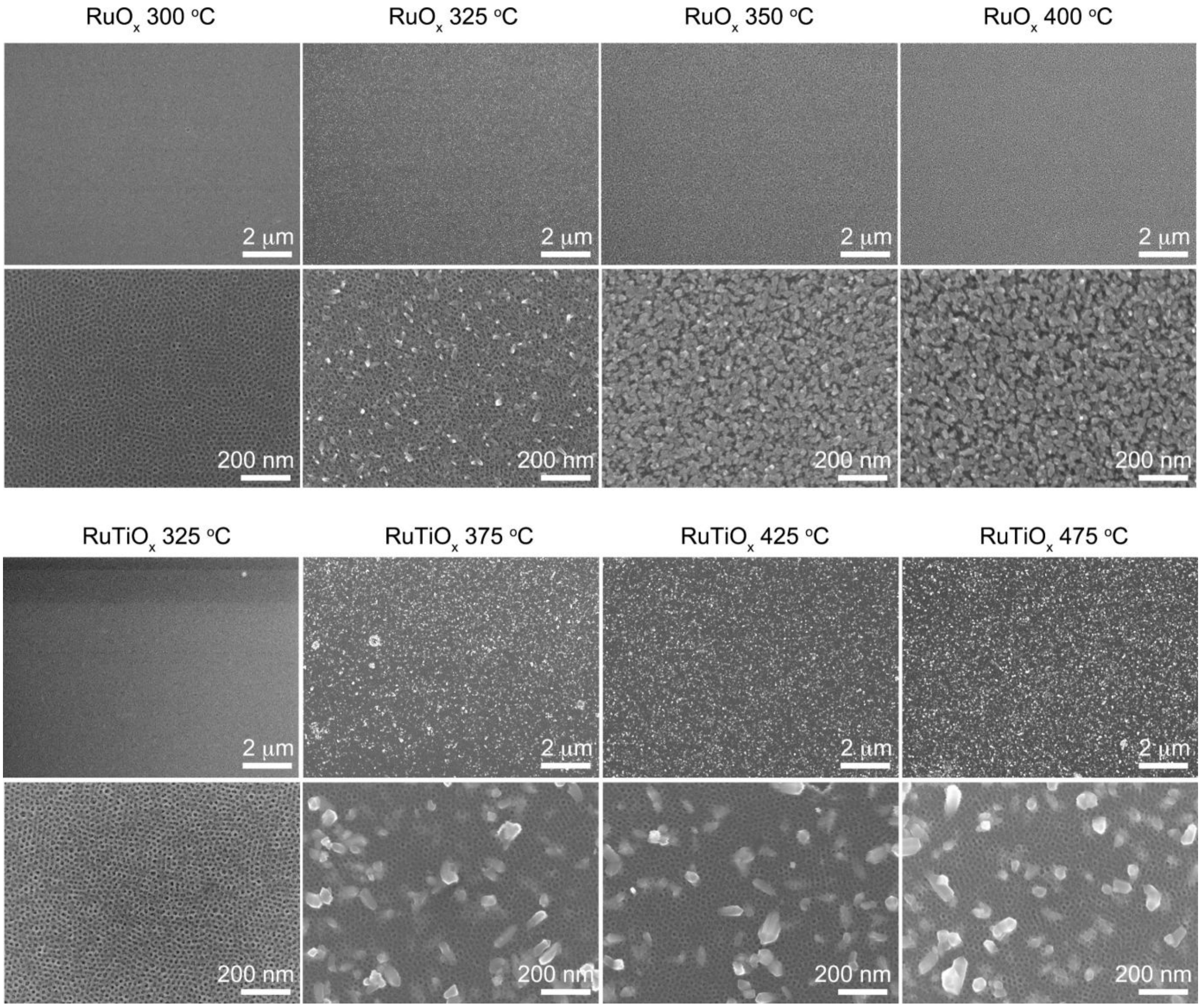

Figure S2: Large scale SEM micrographs of the as-prepared films.

3. Transmission electron microscopy (TEM)

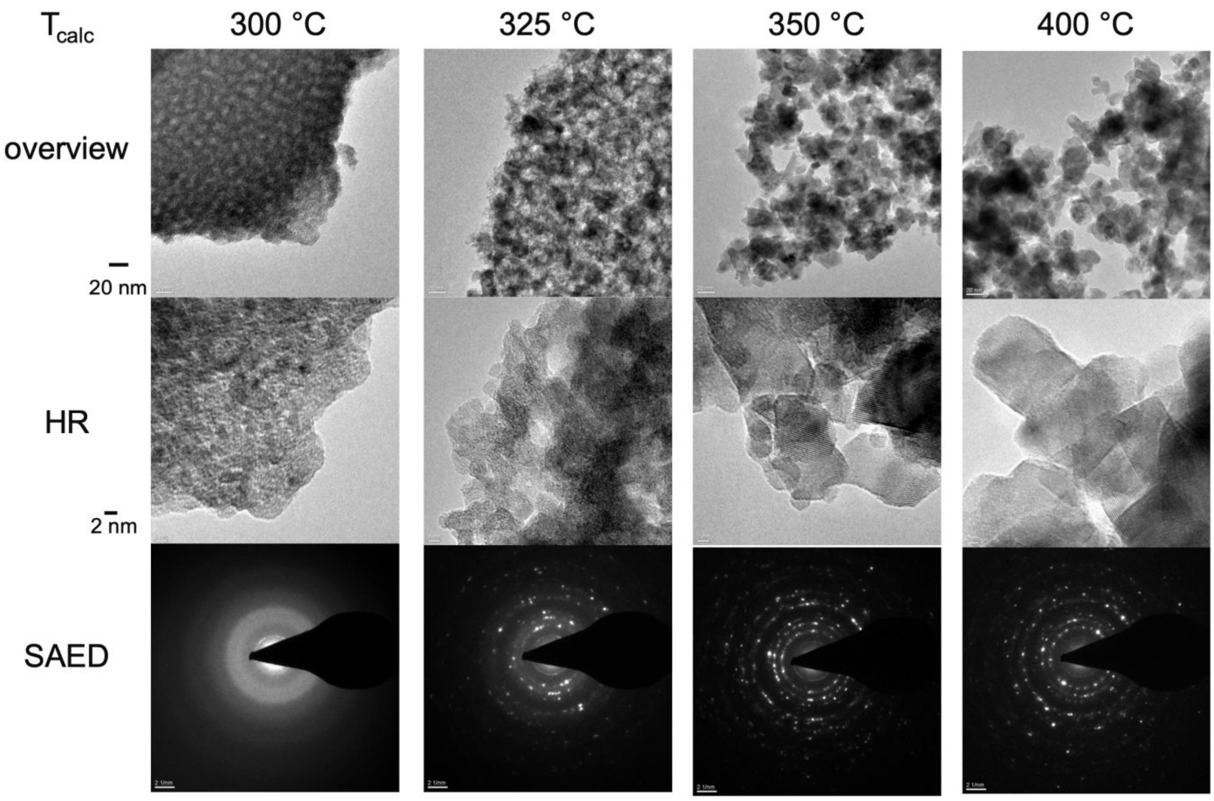

Figure S3: TEM micrographs and SAED pattern of the as-prepared RuO_x films.

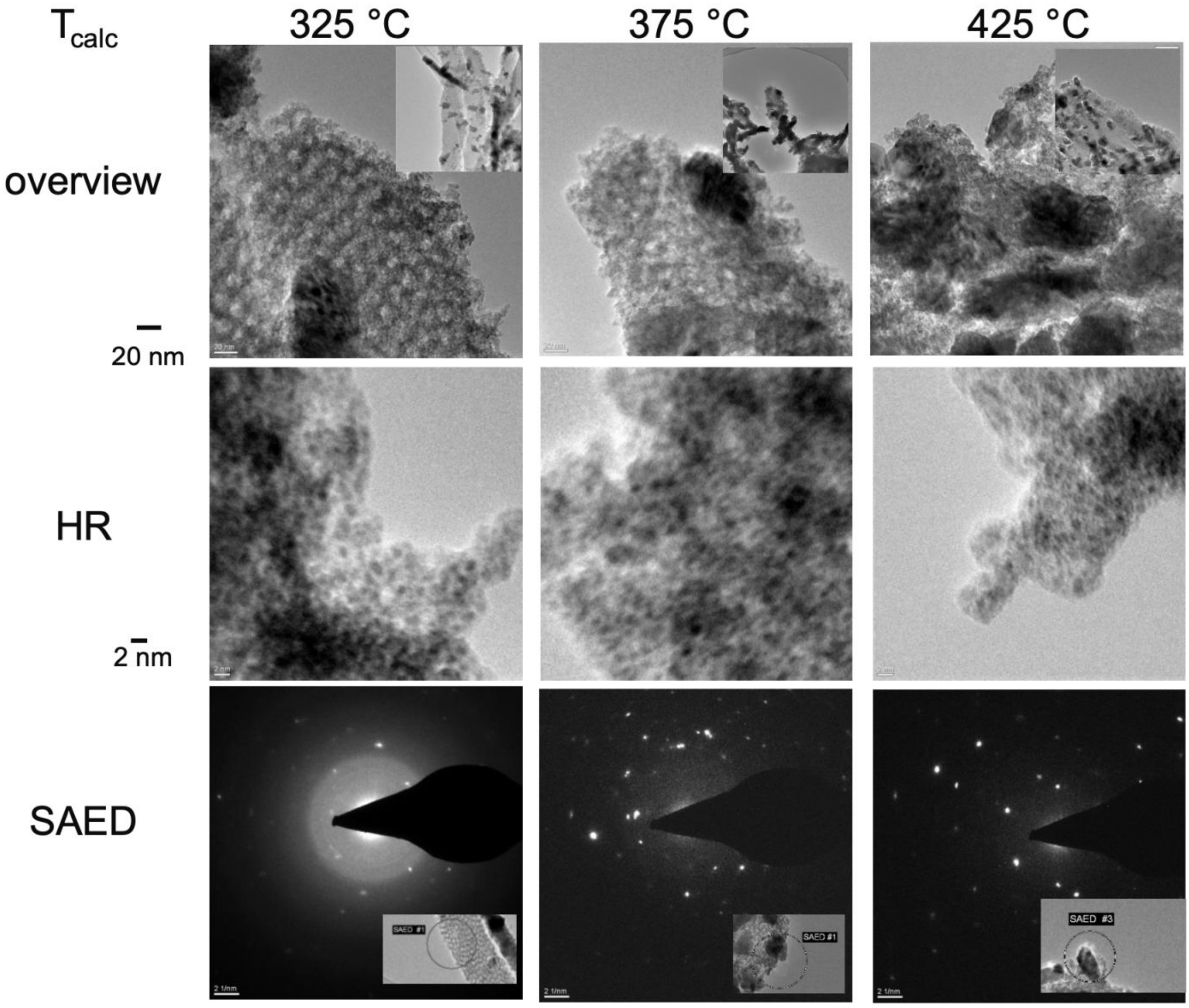

Figure S4: TEM micrographs and SAED pattern of the as-prepared $RuTiO_x$ films.

Transmission electron microscopy was used to study the morphological characteristics of the catalyst films. The HR-TEM images reveal that the mesoporous structure is lost for RuO_x at T>=350 ^{0}C (figure S3) whereas the $RuTiO_x$ films appears to maintain a mesoporous network at high calcination temperatures (figure S4) which can also be seen in the SEM images in figure 1 of the manuscript. For RuO_x, crystallites of size 10-20 nm are formed whereas smaller crystallites of 2-5 nm are observed for $RuTiO_x$. The SAED patterns shows that the crystallinity of both the films increases with the increase in calcination temperature.

4. (Near-)surface composition of $RuTiO_x$ films

Analysis of the Ru 3p : Ti 2p ratio shows that there is some level of inhomogeneity in the $RuTiO_x$ structure. Specifically, we used the, Ru 3p : Ti 2p ratio obtained from XPS to extract the Ru:Ti ratio in the (near-)surface region of the oxide as a function of calcination temperature to observe if calcination induces elemental redistribution. As shown in figure S5, the surface composition is indeed affected by the calcination temperature. This can be at least in part explained by the partial demixing observed in figure 1, which leads to the formation of large RuO_2 crystallites on top of the mesoporous structure. A

significant part of the Ru atoms will be "hidden" in the bulk of these RuO_2 crystallites, obscuring them from the surface sensitive XPS measurements.

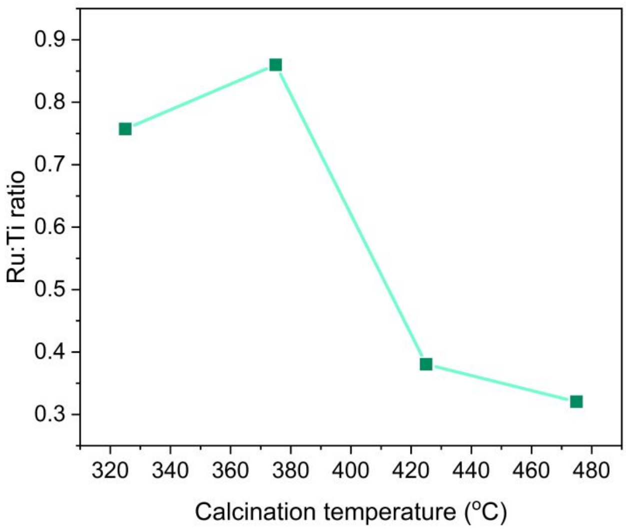

Figure S5: Temperature dependence of the (near-) surface Ru:Ti ratio in the as-prepared $RuTiO_x$ films, derived from the Ru3p:Ti2p ratio measured with XPS with an X-ray energy of 1100 eV.

5. Chloride ions in the lattice

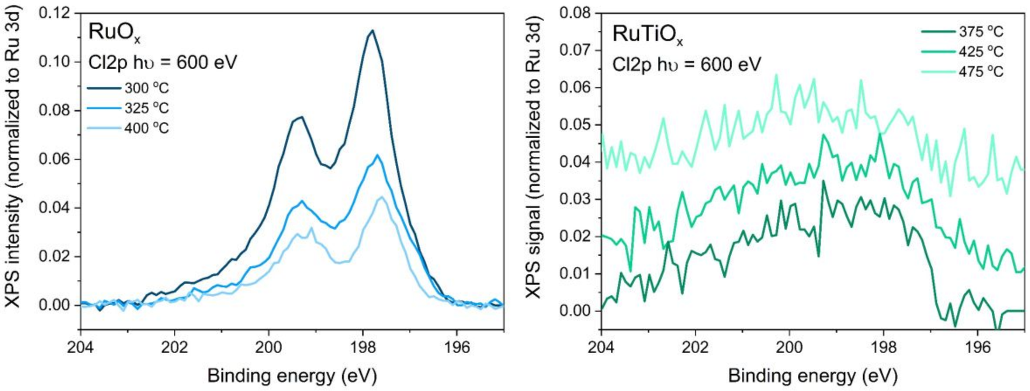

Figure S6: Cl2p XPS spectra of the as-prepared films. The Cl2p peaks were normalized to the area of the Ru3d peak obtained with an excitation energy of 680 eV.

Figure S6 shows that some Cl from the precursor salts remains in the mesoporous films even after calcination. For the RuO_x films, a doublet is observed with a binding energy similar to Cl in $RuCl_3$. For $RuTiO_x$, the Cl2p peak is very broad, suggesting that the Cl is incorporated in the lattice with various coordination environments, i.e. with variation in the Ti and Ru coordination. Note that Cl in TiO_x indeed has a binding energy higher than in RuO_x, as confirmed by Cl2p spectra obtained from mesoporous TiO_x films.

Comparing the Ru 3d normalized intensities of the spectra, and considering the lower Ru-content in $RuTiO_x$, one can observe that the Cl-content of the RuO_x and $RuTiO_x$ for comparable calcination temperature is similar.

6. Electronic structure of the as-prepared films

As discussed in the main text, the electronic structure of the mesoporous films strongly depends on their crystallinity and composition. A first sign for this comes from the sheet conductivity measurements (see figure 3 in the main text). The RuO_x films, in particular the crystalline ones, show metallic conductivity, whereas semi-metallic properties are observed for the $RuTiO_x$. Conductivity in ruthenium oxides is strongly related to the Ru 4d valence electrons[1]. Hence, the differences in conductivity imply differences in the electronic structure of the Ru ions. This is also clearly reflected in the Ru 3d XPS spectra. For Ru oxides, the Ru 3d spectrum typically consists of a main peak doublet and a satellite doublet. This splitting of the Ru 3d spectrum in main peaks and satellites originates from the fact that the emission of Ru3d electrons during the XPS experiments can lead to multiple final states in the ionized Ru atoms that are left behind[2–4]. The main peak is generally identified as the well-screened final state, in which the Ru 4d and/or ligand electrons screen the core hole. The satellite peaks are identified as the poorly screened final state, in which there is little response of the valence electrons to the presence of the core hole during the photo-ionization process. Since very conductive Ru oxides have a high density of Ru 4d states around the Fermi level[1], their ability to screen the core hole is generally good. Hence, this leads to a pronounced screened main line at low binding energy and a relatively weak, but well-separated satellite. This is the case for rutile RuO_2. For Ru oxides with lower conductivity, screening will be less effective. This can lead to very strong satellite peak intensity and/or a broad, unresolved main line/satellite structure, as is observed for the $RuTiO_x$ films and amorphous RuO_x.

7. O K-edge XAS

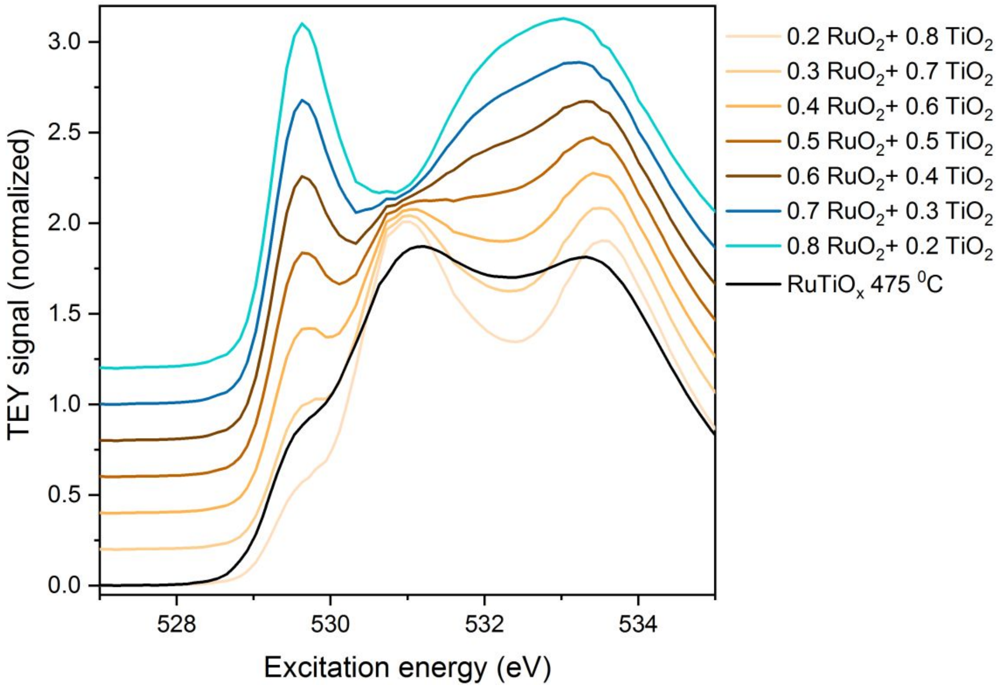

Figure S7: Linear combination of the O K-edge spectra of RuO_2 450 °C and TiO_2 475 °C compared to $RuTiO_x$.

The O K-edge absorption spectrum of $RuTiO_x$ (figure 4 in the main text) shows three distinct features at 529.6 eV (A), 530.9 eV (B), and 533.5 eV (C). The feature A overlaps with O K-edge spectra of RuO_2 and corresponds to μ_3-O oxygen atoms of RuO_x lattice[5]. The features B and C overlap with those of TiO_2, corresponding to electronic transitions from the O 1s orbital to the hybridized O 2p+Ti 3d t_{2g} orbitals and O 2p+Ti 3d e_g orbitals, respectively[6,7]. However, at a quantitative level, the $RuTiO_x$ is not a linear combination of the pure TiO_2 (anatase) and RuO_2 450 ^{0}C spectra, as shown in figure S7. This implies that the experimentally obtained spectra is not from two different materials (RuO_2 and TiO_2) but from a newly formed $RuTiO_x$ phase. More specifically, it can be observed that the Ru-O signal is relatively weak (not consistent with the amount or Ru-O present at the surface according to figure 5 in the main text), and the Ti-O features are broadened.

8. Carbon content

To analyze the carbon content of the samples, we determined the C1s/(Ru3d+C1s) ratio from our XPS data. The results are summarized below in table S1. It should be noted that due to the lineshape/satellite change in the Ru 3d spectra with increasing calcination temperature, there is some uncertainty margin in the analysis, causing some scatter in the data. Compared to the uncalcined samples, it is clear that most carbon is removed during the calcination. Much of the remnants that remain are quite persistent up to high temperatures, suggesting that they may be trapped in the grain boundaries of the oxide, potentially functioning as a binder. Of note, the carbon content of the RuO_x and $RuTiO_x$ samples is fairly similar.

Table S1: XPS analysis of carbon content of the prepared catalyst

**Material**	**T calcination ^{0}C**	**C1s/(C1s+Ru3d)**
RuO_x	Uncalcined	0.51
	325	0.14
	375	0.12
	400	0.20
$RuTiO_x$	Uncalcined	0.60
	325	0.18
	375	0.24
	425	0.19
	475	0.17

9. Comparison of Chlorine evolution performance

Table S2: Literature comparison of the performance of chlorine evolution catalysts

**Catalyst**	**Electrolyte**	**Electrode potential @ 10 mA cm^{-2}**	**Stability (time & conditions)**	**Reference**
$RuTiO_x$ 425 °C	3M HCl	1.432 V_{RHE}	4 hours @ 100 mA/cm^2	This work

RuO_x 400 °C	3M HCl	1.437 V_{RHE}	4 hours @ 100 mA/cm^2	This work
RuO_2@TiO_2/TP	0.5M NaCl + 0.01M $HClO_4$	1.70 V_{RHE}	24 hours @ 50 mA/cm^2	Supplementary ref[8]
RuO_2-TiO_2	5M NaCl + 0.001M $HClO_4$	1.50 V_{RHE}	2 hours @ 1 A/cm^2	Supplementary ref[9]
Ru_1-Ti_4O_7	5M NaCl	1.507 V_{RHE}	100 hours @ 100 mA/cm^2	Supplementary ref[10]
$(Ru_{0.3}Ti_{0.7})$-TNTs	5M NaCl	1.618 V_{RHE}		Supplementary ref[11]
Commercial DSA, $Ru_{0.3}Ti_{0.7}O2$ (Covestro, Germany)	3.5M NaCl	1.674 V_{RHE}		Supplementary ref[12]
Ru-Ir/TiO_2	4M NaCl + 0.001M $HClO_4$	1.65 V_{RHE}		Supplementary ref[13]
Ru_1-TiO_x	4M NaCl	1.54 V_{RHE}		Supplementary ref[14]

References:

(1) Cox, P. A.; Egdell, R. G.; Goodenough, J. B.; Hamnett, A.; Naish, C. C. The Metal-to-Semiconductor Transition in Ternary Ruthenium (IV) Oxides: A Study by Electron Spectroscopy. *J. Phys. C Solid State Phys.* **1983**, *16* (32). https://doi.org/10.1088/0022-3719/16/32/014.

(2) Cox, P. A.; Goodenough, J. B.; Tavener, P. J.; Telles, D.; Egdell, R. G. The Electronic Structure of Bi2-XGdxRu2O7 and RuO2: A Study by Electron Spectroscopy. *J. Solid State Chem.* **1986**, *62* (3). https://doi.org/10.1016/0022-4596(86)90251-3.

(3) Kim, Y. J.; Gao, Y.; Chambers, S. A. Core-Level X-Ray Photoelectron Spectra and X-Ray Photoelectron Diffraction of RuO 2 (110) Grown by Molecular Beam Epitaxy on TiO 2 (110). *Appl. Surf. Sci.* **1997**, *120* (3–4). https://doi.org/10.1016/S0169-4332(97)00233-X.

(4) Morgan, D. J. Resolving Ruthenium: XPS Studies of Common Ruthenium Materials. *Surf. Interface Anal.* **2015**, *47* (11), 1072–1079. https://doi.org/10.1002/sia.5852.

(5) Deka, N.; Jones, T. E.; Falling, L. J.; Sandoval-Diaz, L. E.; Lunkenbein, T.; Velasco-Velez, J. J.; Chan, T. S.; Chuang, C. H.; Knop-Gericke, A.; Mom, R. V. On the Operando Structure of Ruthenium Oxides during the Oxygen Evolution Reaction in Acidic Media. *ACS Catal.* **2023**, *13* (11). https://doi.org/10.1021/acscatal.3c01607.

(6) Park, S. H.; Katoch, A.; Chae, K. H.; Gautam, S.; Miedema, P.; Cho, S. W.; Kim, M.; Wang, R. P.; Lazemi, M.; de Groot, F.; Kwon, S. Direct and Real-Time Observation of Hole Transport Dynamics in Anatase TiO2 Using X-Ray Free-Electron Laser. *Nat. Commun.* **2022**, *13* (1). https://doi.org/10.1038/s41467-022-30336-1.

(7) Xue, J.; Zhu, X.; Zhang, Y.; Wang, W.; Xie, W.; Zhou, J.; Bao, J.; Luo, Y.; Gao, X.; Wang, Y.; Jang, L. Y.; Sun, S.; Gao, C. Nature of Conduction Band Tailing in Hydrogenated Titanium Dioxide for Photocatalytic Hydrogen Evolution. *ChemCatChem*. 2016.

https://doi.org/10.1002/cctc.201600628.

(8) Zhang, L.; Liang, J.; He, X.; Yang, Q.; Luo, Y.; Zheng, D.; Sun, S.; Zhang, J.; Yan, H.; Ying, B.; Guo, X.; Sun, X. Integrating RuO2@TiO2 Catalyzed Electrochemical Chlorine Evolution with a NO Oxidation Reaction for Nitrate Synthesis. *Inorg. Chem. Front.* **2023**, *10* (7). https://doi.org/10.1039/d3qi00209h.

(9) Xiong, K.; Peng, L.; Wang, Y.; Liu, L.; Deng, Z.; Li, L.; Wei, Z. In Situ Growth of RuO2–TiO2 Catalyst with Flower-like Morphologies on the Ti Substrate as a Binder-Free Integrated Anode for Chlorine Evolution. *J. Appl. Electrochem.* **2016**, *46* (8). https://doi.org/10.1007/s10800-016-0934-4.

(10) Lee, W.; Choung, S.; Kim, S.; Hong, J.; Kim, D.; Tarpeh, W. A.; Han, J. W.; Cho, K. Atomically Dispersed Ru-Doped Ti4O7 Electrocatalysts for Chlorine Evolution Reaction with a Universal Activity. *Small* **2024**, *20* (35), 2401248. https://doi.org/https://doi.org/10.1002/smll.202401248.

(11) Xiong, K.; Deng, Z.; Li, L.; Chen, S.; Xia, M.; Zhang, L.; Qi, X.; Ding, W.; Tan, S.; Wei, Z. Sn and Sb Co-Doped RuTi Oxides Supported on TiO2 Nanotubes Anode for Selectivity toward Electrocatalytic Chlorine Evolution. *J. Appl. Electrochem.* **2013**, *43* (8). https://doi.org/10.1007/s10800-013-0570-1.

(12) Chen, R.; Trieu, V.; Zeradjanin, A. R.; Natter, H.; Teschner, D.; Kintrup, J.; Bulan, A.; Schuhmann, W.; Hempelmann, R. Microstructural Impact of Anodic Coatings on the Electrochemical Chlorine Evolution Reaction. *Phys. Chem. Chem. Phys.* **2012**, *14* (20). https://doi.org/10.1039/c2cp41163f.

(13) Menzel, N.; Ortel, E.; Mette, K.; Kraehnert, R.; Strasser, P. Dimensionally Stable Ru/Ir/TiO2-Anodes with Tailored Mesoporosity for Efficient Electrochemical Chlorine Evolution. *ACS Catal.* **2013**, *3* (6). https://doi.org/10.1021/cs4000238.

(14) Yao, Y.; Zhao, L.; Dai, J.; Wang, J.; Fang, C.; Zhan, G.; Zheng, Q.; Hou, W.; Zhang, L. Single Atom Ru Monolithic Electrode for Efficient Chlorine Evolution and Nitrate Reduction. *Angew. Chemie - Int. Ed.* **2022**, *61* (41). https://doi.org/10.1002/anie.202208215.
